# Carotenoids and risk of fracture: a meta-analysis of observational studies

**DOI:** 10.18632/oncotarget.13678

**Published:** 2016-11-29

**Authors:** Jiuhong Xu, Chunli Song, Xiaochao Song, Xi Zhang, Xinli Li

**Affiliations:** ^1^ Department of Radiotherapy, The First Affiliated Hospital of Soochow University, Suzhou Jiangsu, PR China, 215006; ^2^ School of Public Health, Medical College of Soochow University, Suzhou, Jiangsu, PR China, 215123; ^3^ Clinical Research Unit, Xinhua Hospital Affiliated to Shanghai Jiaotong University School of Medicine, Shanghai, PR China, 200092; ^4^ Jiangsu Key Laboratory of Preventive and Translational Medicine for Geriatric Diseases, School of Public Health, Soochow University, Suzhou, PR China, 215123

**Keywords:** carotenoids, carotene, lycopene, β-cryptoxanthin, lutein/zeaxanthin

## Abstract

To quantify the association between dietary and circulating carotenoids and fracture risk, a meta-analysis was conducted by searching MEDLINE and EMBASE databases for eligible articles published before May 2016. Five prospective and 2 case-control studies with 140,265 participants and 4,324 cases were identified in our meta-analysis. Among which 5 studies assessed the association between dietary carotenoids levels and hip fracture risk, 2 studies focused on the association between circulating carotenoids levels and any fracture risk. A random-effects model was employed to summarize the risk estimations and their 95% confidence intervals (CIs). Hip fracture risk among participants with high dietary total carotenoids intake was 28% lower than that in participants with low dietary total carotenoids (OR: 0.72; 95% CI: 0.51, 1.01). A similar risk of hip fracture was found for β-carotene based on 5 studies, the summarized OR for high vs. low dietary β-carotene was 0.72 (95% CI: 0.54, 0.95). However, a significant between-study heterogeneity was found (total carotene: I^2^ = 59.4%, *P* = 0.06; β-carotene: I^2^ = 74.4%, *P* = 0.04). Other individual carotenoids did not show significant associations with hip fracture risk. Circulating carotene levels had no significant association with any fracture risk, the pooled OR (95% CI) was 0.83 (0.59, 1.17). Based on the evidence from observational studies, our meta-analysis supported the hypothesis that higher dietary total carotenoids or β-carotene intake might be potentially associated with a low risk of hip fracture, however, future well-designed prospective cohort studies and randomized controlled trials are warranted to specify the associations between carotenoids and fracture.

## INTRODUCTION

Osteoporosis, characterized by obvious bone loss and micro-architectural disruption, results in bone fragility and an increased susceptibility to fractures [1]. Elderly population, especially older women, are at higher risk of osteoporosis, as aging could facilitate the decrease of bone mass [2]. With the increase of the ratio of elderly people, osteoporosis and osteoporotic fracture is now regarded to be an age-related serious public health problem worldwide.

Experimental research has highlighted the involvement of reactive oxygen species (ROS) and free radicals in inducing bone loss by regulating osteoclastogenesis [3] and osteoclastic differentiation [4], apoptosis of osteoblasts and osteocytes [5], and decreasing osteoblastic differentiation [6]. Epidemiological studies also suggested a negative association between oxidative stress and BMD (Bone Mineral Density) [7–9], or risk of osteoporosis, which is further supported by the evidence of elevated serum oxidative stress marker in osteoporosis patients [10]. Thus, it is reasonable to make a hypothesis that inhibition of oxidative stress might be useful to counteract the decrease of BMD, slow down the process of osteoporosis, and reduce the risk of osteoporotic fractures.

Dietary antioxidant carotenoids, enriched in vegetables and fruits, have been testified to protect the human body's defense against the reactive oxygen species [11]. Carotenoids mainly include β-carotene, α-carotene, β-cryptoxanthin, lutein, zeaxanthin, and lycopene, which account for ~70% of all carotenoids. Those individual carotenoids had different chemical formula, molecular mass and structure, antioxidant ability, and also conserve different ability to convert into Vitamin A. Although the results from animal studies suggested that lycopene [12, 13] and β-cryptoxanthin [14] could reduce the risk of osteoporosis and related fractures, epidemiological studies have drawn inconsistent conclusions. Several studies, including the Framingham Osteoporosis Study [15] and the Utah Study of Nutrition and Bone Health [16], reported that high intake or high serum levels of carotenoids was associated with decreased risk of osteoporosis and fracture [10, 17–24], while other researches deduced conflicting results, Barker et al. found a non-significant association between serum β-carotene and hip fracture risk among elderly women [25], Framingham Osteoporosis Study also showed a null association of dietary lycopene with hip fracture risk among women [15].

Thus, our present work aimed to conduct a quantitative meta-analysis by summarizing the evidence from current observational studies to comprehensively clarify the associations between carotenoids and fractures risk.

## RESULTS

### Literature collection

**Figure 1:**
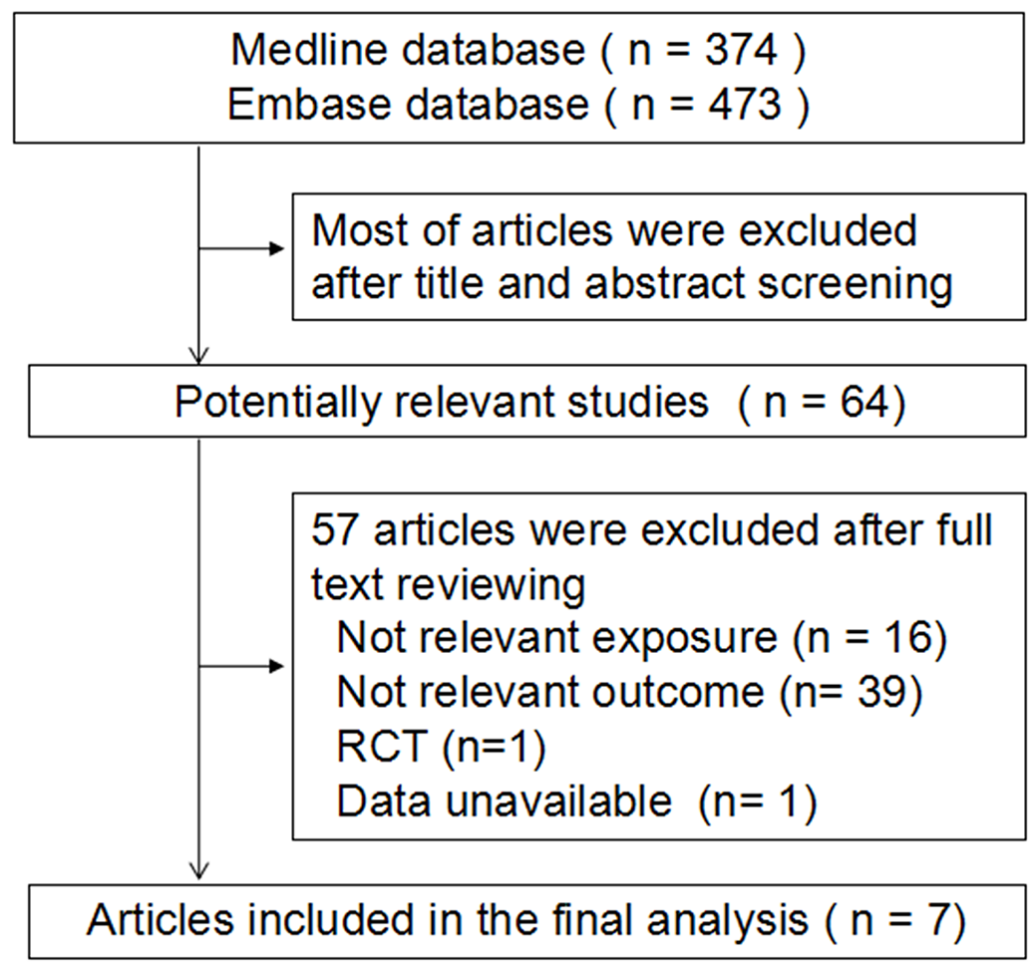
Flow chart of literature search

We identified 374 articles from the MEDLINE database and 473 from EMBASE database. After excluding the review papers, papers without reporting risk estimates, papers with irrelevant exposure factors (mainly related to Vitamin A, retinol or dietary pattern), papers with irrelevant endpoints (mainly about BMD or bone turnover), or basic experimental research by title/abstract screening, a total of 64 articles were selected for full-text review. We finally identified 7 studies by using full-text screening, including 5 prospective [15, 22, 23, 25, 26] and 2 case-control studies [16, 27]. Five studies [15, 16, 23, 26, 27] assessed the association between dietary carotenoids levels and hip fracture risk and 2 studies [22, 25] focused on the association between circulating carotenoids levels and risk of any fracture. The detailed process for study section is presented in Figure 1.

### Characteristics of the included studies

The characteristics of all 7 selected studies are displayed in Table 1. These studies were published between 2002 and 2014, with a total of 140,265 participants and 4,324 fracture cases. All participants of these studies aged ≥ 50 years (range: 50–80 years) and their mean BMI ranged from 21.5 to 27.4 kg/m^2^. Nearly 80% (111,336/140,265) of studies participants were women and all women were post-menopausal. Two studies were conducted among Asian population [23, 27], 3 studies were conducted among Americans [15, 16, 26], and 2 studies were in Oceania [22] and Europe [25], respectively. The follow-up duration of prospective studies ranged from 3.7 years to 18 years. Dietary carotenoids intake was assessed by validated food frequency questionnaires (FFQ), and circulating carotenoids concentrations were determined by the high-performance liquid chromatography (HPLC). All fracture cases were ascertained by surgical records or medical records except Ambrosini's study [22], in which fracture cases were self-reported. The variables adjusted in the regression models mainly included age, BMI, education, total energy intake, smoking status, physical activity, intakes of calcium, soy isoflavones and vitamin B6, menopausal status, use of hormone replacement therapy, and history of diabetes and stroke.

**Table 1:** Characteristics of selected studies in the meta-analysis

Author^(ref)^/ year/location	Study Design/Duration	Case/participants/female,%/age/ BMI, kg/m^2^	Source/special CA	CA assessment/compared groups	Research endpoint/Case ascertainment	Adjusted variables	Quality Score
Dai^(23)^ /2014 /Singapore	Cohort /9.9 y	1630/61524/ 55.8%/56.3y/men: 23; women:23.2	Diet/total CA; α-,β- carotene, lycopene, β-cryptoxanthin, lutein/zeaxanthin	FFQ/α-carotene:Q1 < 59.8;Q4 > 212.3; β-carotene: Q1 < 850.4; Q4 > 1772.4; lutein/zeaxanthin:Q1 < 781.8; Q4 > 1544.1; Lycopene: Q1 < 191.9; Q4 > 858.4	Hip fracture /Surgical or medical records	Age, recruitment year, dialect group, BMI, education, total energy intake, smoking, physical activity, calcium, vitamin B6, soy isoflavones, menopausal status, history of diabetes and stroke, use of HRT.	8
Sahni^(15)^ /2009 /US	Cohort /17 y	100/946/61%/75y /25.5	Diet/total CA; α-,β- carotene, lycopene, β-cryptoxanthin, lutein/zeaxanthin	FFQ/carotenoids; T1: 7299; T3:23711 Lycopene: T1:2710; T3:12664	Hip fracture /Medical records, radiographic and operative reports	Sex, age, BMI, height, energy intake, physical activity, alcohol intake, smoking, calcium intake, vitamin D intake, caffeine intake	8
Feskanich^(26)^ /2002 /US	Cohort /18 y	603/72337/100%/59.6y/26	Diet /β-carotene	FFQ/β-carotene Q1: < 2550; Q5:≥ 6300	Hip fracture /Self-report, and medical record confirmed	Age, follow-up cycle, BMI, HRT, smoking, physical activity, use of thiazide diuretics, intake of calcium, protein, Vitamin D, Vitamin K, alcohol, caffeine.	8
Sun^(27)^ /2014 /China	CC /NR	726/1452/75.6%/70.9y/control:23.1; case: 21.5	Diet /β-carotene	FFQ/β-Carotene male:Q1:1882; Q4: 5954; female:Q1:1622; Q4: 6281	Hip fracture /Medical records	Age, sex, drugs, BMI, education, occupation, household income, family history, smoking, alcohol drinking, Ca and multivitamin supplementation, physical activity, intake of energy and selected nutrients.	7
Zhang^(16)^ /2005 /US	CC /NR	835/1826/78.5% /75.8y/control: 26.4; case: 24.5	Diet /β-carotene	FFQ/β-Carotene Q1: 1.8; Q5:12.2	Hip fracture /Medical records	Age, sex, BMI, physical activity, energy intake, protein, caffeine and alcohol, calcium and vitamin D intakes.	7
Ambrosini^(22)^ /2014, /Australia	Prospective /7.0 y	123/929/ 33.6%/50.8y/control: 28.1; case: 27.4	Plasma /carotene	HPLC/carotene T1: 0.1-0.6; T3: 1.2-16.7	Any fracture/Self-report	Sex, age, medications, previous fracture, smoking status	7
Barker^(25)^ /2005 /British	NCC /3.7 y	312/1246/100% /80y/NR	Serum /β-carotene	HPLC/β-Carotene NR	Hip fracture and any fracture/Medical records	Age, serum 25(OH)D, βCTX, bone ALP, total hip BMD, weight, height, smoking, exercise, milk consumption	7

BMI: Body Mass Index; CA: carotenoids; FFQ: Food Frequency Questionnaire; HRT: Hormone Replace Therapy; CC: Case-Control study; NR: Not Reported; NCC: Nested Case-Control; HPLC: High-performance liquid chromatography; β-CTX: β Crosslaps; ALP: Alkaline Phosphatase; BMD: Bone Mineral Density.

### Dietary total or individual carotenoids and hip fracture risk

**Figure 2:**
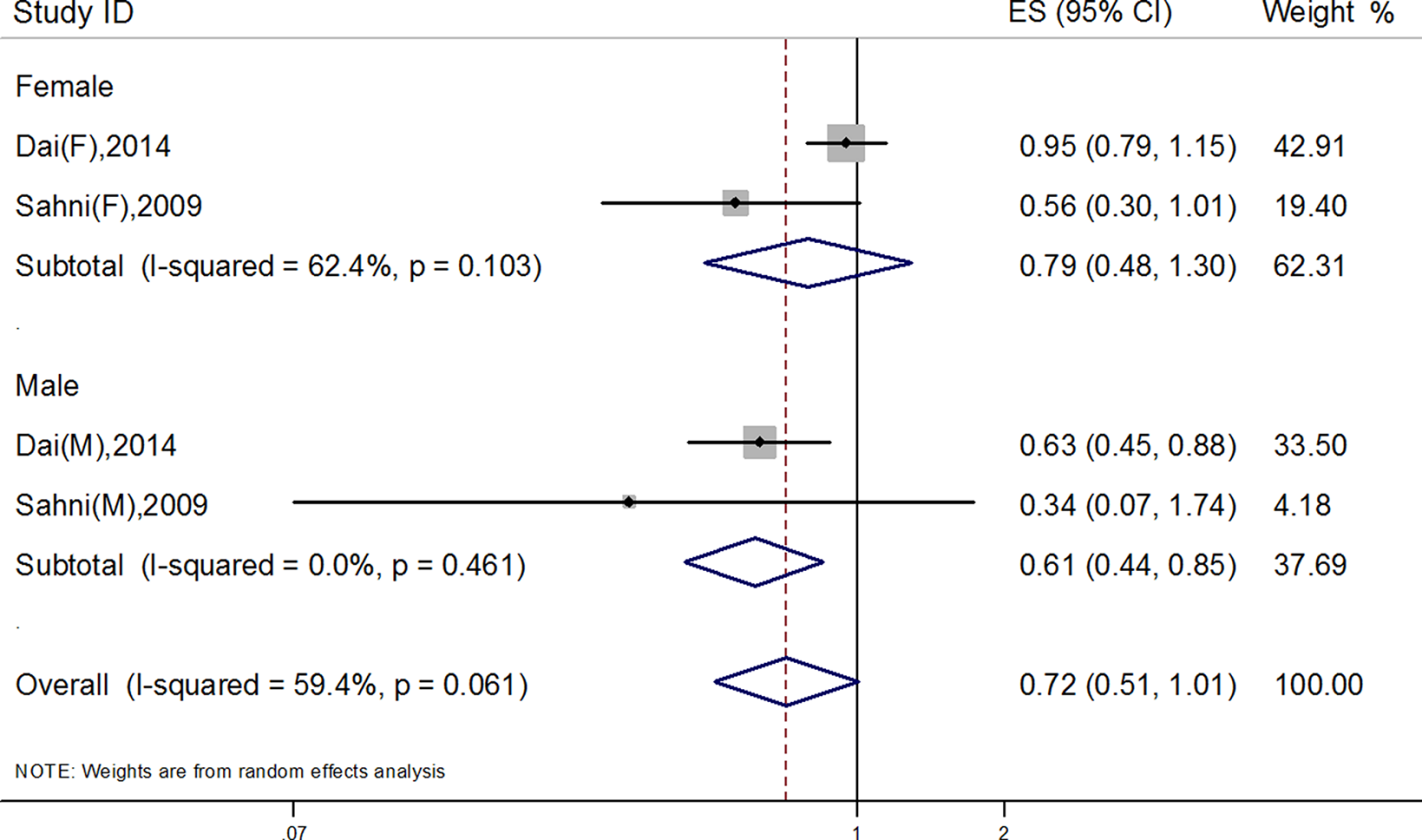
Association of total dietary carotenoids and hip fracture risk

Of all 7 identified studies, 5 studies focused on the association between dietary carotenoids and hip fracture risk, among which 2 studies focused on the dietary total carotenids. Compared to the participants with low carotenoids intake, the OR of hip fracture risk among participants with high total carotenoids intake was 0.72 (95% CI: 0.51, 1.01; I^2^ = 59.4%, *P* = 0.06) (Figure 2).

**Figure 3:**
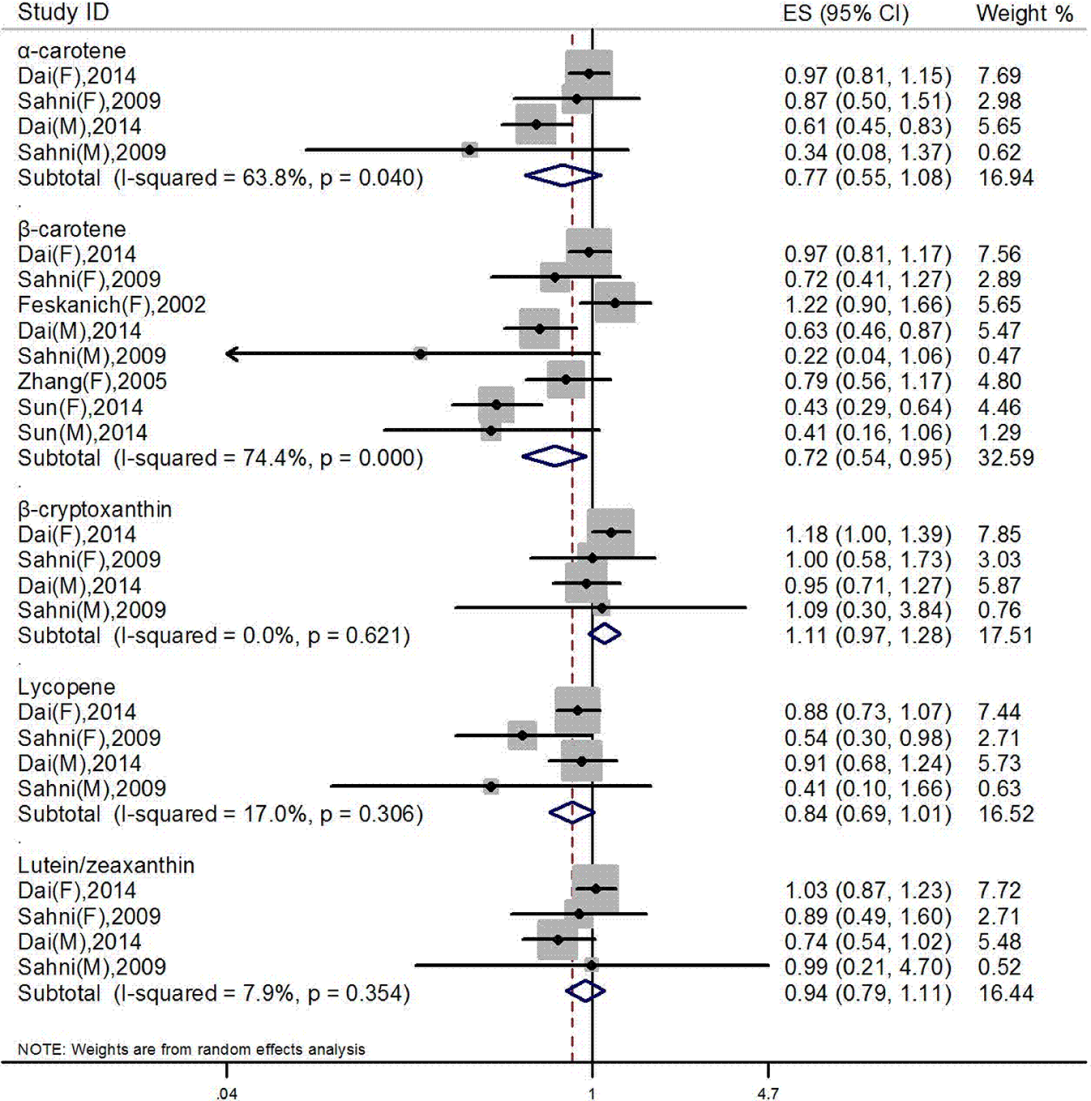
Pooled analysis between dietary special carotenoids and hip fracture risk

Five studies explored the relationship between dietary individual carotenoids, such as α-, β-carotene, β-cryptoxanthin, lycopene, and Lutein/zeaxanthin, and hip fracture risk. As shown in Figure 3, high intake of dietary β-carotene significantly decreased the risk of hip fracture, and the OR was 0.72 (95% CI: 0.54, 0.95), while other individual carotenoids did not show any significant associations with hip fracture risk, the pooled OR was 0.77 (95% CI: 0.55, 1.08) for α-carotene, 1.11 (95% CI: 0.97, 1.28) for β-cryptoxanthin, 0.84 (95% CI: 0.69, 1.01) for lycopene, and 0.94 (95% CI: 0.79, 1.11) for Lutein/zeaxanthin, respectively. Significant heterogeneities were found in the meta-analyses of α-carotene (I^2^ = 63.8%, *P* < 0.001) and β-carotene (I^2^ = 74.4%, *P* = 0.04), respectively.

Further subgroup analysis showed that dietary β-carotene decreased hip fracture risk among men (OR = 0.57, 95% CI: 0.40, 0.81) or subjects aged over 60 years (OR = 0.57, 95% CI: 0.40, 0.81), or subjects with BMI ≤ 25 kg/m^2^ (OR = 0.62, 95% CI: 0.40, 0.96, *P* = 0.03), or Asians (OR = 0.62, 95% CI: 0.40, 0.96, *P* = 0.03), while further meta-regression analysis showed that the study characteristics of gender, age, BMI and location did not affect our results. (Table 2).

**Table 2:** Effects of study characteristics on the association between dietary β-carotene and hip fracture risk

Group	No. of studies	OR (95% CI)	*P*_interaction_
**All**	8	0.72 (0.54, 0.95)	
**Study design**			0.21
Prospective	5	0.84 (0.62, 1.14)	
Case-control	3	**0.55 (0.34, 0.89)**	
**Gender**			0.22
Female	5	0.80 (0.58, 1.11)	
Male	3	**0.57 (0.40, 0.81)**	
**Age(years)**			0.11
<60	3	0.91 (0.66, 1.27)	
≥60	5	**0.57 (0.40, 0.81)**	
**BMI (kg/m^2^)**			0.40
≤25	4	**0.62 (0.40, 0.96)**	
>25	4	0.85 (0.57, 1.26)	
**Location**			0.40
Asia	4	**0.62 (0.40, 0.96)**	
United States	4	0.85 (0.57, 1.26)	

### Circulating carotene and any fracture risk

There was no study concerning on the association between total circulating carotenoids and fracture risk. Only 2 studies prospectively assessed the relationship between circulating carotene levels and risk of any fracture; the pooled OR for high versus low carotene levels was 0.83 (95% CI: 0.59, 1.17).

### Sensitivity analysis

Results of sensitivity analyses conducted by omitting one study at each time using a random-effects model showed that the combined results for dietary intake of total carotenoids and α-carotene were sensitive to individual study. Dai et al's study focused on female subjects affected the association between dietary total carotenoids, α-carotene and hip fracture risk, and the summary OR was 0.60 (95% CI: 0.45, 0.80) for total carotenoids and 0.65 (95% CI: 0.50, 0.85) for α-carotene respectively when deleted this data set.

### Publication bias

The Egger's tests did not indicate significant publication bias in the meta-analyses for dietary total carotenoids, α-carotene, β-carotene, β-cryptoxanthin, lycopene, or Lutein/zeaxanthin (*P* = 0.16; *P* = 0.36; *P* = 0.10; *P* = 0. 49; *P* = 0.14; *P* = 0.60).

## DISCUSSION

Based on the limited evidence from observational studies, our meta-analysis supported the hypothesis that higher dietary intake of total carotenoids or β-carotene might be potentially associated with a low risk of hip fracture, although an obvious heterogeneity was observed.

The mechanisms that carotenoids decreased hip fracture risk might be multi-factors, one was its antioxidant property [28], combining with its anti-catabolic and pro-anabolic activities, which could suppress osteoclast differentiation, promote osteoblast mineralization and stimulate alkaline phosphatase activity of osteoblasts [13, 29, 30], on the other hand, carotenoids were the index of fruit and vegetable intake, whose beneficial role in decreasing fracture risk also had been widely proved, this support the beneficial role of dietary total carotenoids and β-carotene in decreased hip fracture risk.

Meanwhile, sex hormones regulated the biological effects of carotenoids, testosterone could increase the uptake and biological availability of carotenoids [31], the decreased level of testosterone and the loss of the estrogen function increased the risk of fracture. In our present study, the association of dietary intake of β-carotene with decreased fracture risk for men might be the synergistic effects of testosterone and the antioxidant property of β-carotene, while for postmenopausal women, the effect of oxidative stress on bone health may be less critical than estrogen level. Older people conserved higher susceptibility to fracture as aging resulted in serious bone loss [2], continually decreased level of estrogen and testosterone also increased hip fracture risk, thus, the positive association of dietary β-carotene intake with bone health might be more pronounced for those subjects who were at higher risk of fracture.

Previous research showed that BMI < 20 kg/m^2^ is an independent risk factor of fracture [32], as low BMI usually encountered increased oxidative damage [33], while a higher BMI is regarded as a protective factor against hip fractures for the greater weight bearing in the loading bone sites [34], and the increased endogenous estrogen produced in adipose tissues for postmenopausal women [35]. In our present study, β-carotene intake was associated with decreased hip fracture risk for subjects with BMI < 25 kg/m^2^, this might be explained by the antioxidant property of carotenoids, on the other hand, subjects with lower BMI had less adipose tissue to store carotenoids, and the effects of carotenoids could be more direct.

Population came from different geographic area shared different genetic background, different dietary habit and lifestyle, which might result in the difference in the intake of carotenoids. Except the fact that Asian population have relatively lower rates of hip fracture [36] and shorter hip axis length [37], the significant association of β-carotene intake with decreased hip fracture risk for Asians might be the higher intake of vegetables and fruits, which was the main source of carotenoids in Asia [23], and their beneficial effect on fractures had been wildly testified [13, 15, 18, 27, 38]. Meanwhile, this also could explain the positive association of β-carotene with decreased hip fracture for subjects with BMI < 25 kg/m^2^, as those subjects were all Asians.

Although there had many factors affected the absorption and tissue distribution of dietary carotenoids, blood is always the main tissue for carotenoids distribution [39]. Results from carefully controlled human intervention trials also testified the positive association between circulating carotenoids concentrations and dietary intake [39, 40], while the summary results of circulating carotene and fracture risk was inconsistent with the role of dietary carotene intake. The relatively small number of sample, measurement error or misclassification of exposure in assessing dietary carotenoids intake from a dietary questionnaire could result in the discrepancy between dietary and circulating level of carotenoids [41], on the other hand, the effects of carotenoids on fracture might be related to the subtype of fracture.

Our meta-analysis has limitations. First, there were not sufficient studies included, which attenuated the statistical power to detect the minor difference, limited our further dose-response meta-analysis, and this was also the common reason of heterogeneity. Second, all the included studies did not share the uniform exposure category and reference category of risk estimates, and we extracted the pooled risk estimates for the highest *vs.* the lowest category regardless of their cutoffs. Third, there might exist misclassification of dietary carotenoids intake and fracture assessment. Except one prospective study used repeated measurements of diet and supplement use during follow-up [26], others only used the baseline dietary data to calculate dietary carotenoids intake, and did not adjust for secular changes in diet during the follow-up period, the estimated intake levels might not reflect the actual amount of dietary carotenoids intake. Self-reported fractures might induce misclassification, however, Ivers's study regarded self-report fracture as a reasonable and accurate method for ascertaining the events of fractures in older adults during a long follow-up period [42]. Fourth, observational studies were not powerful to make a casual relationship, and for case-control studies, recall bias and selection bias were common shortcomings. Finally, there existed other residual confounders, such as vitamin D levels and hormone therapy for women.

Our meta-analysis based on 7 observational studies found that dietary intake of total carotenoids and β-carotene conserved the potentiality of decreased hip fracture risk, especially the negative association between dietary β-carotene intake and hip fracture risk for males or subjects aged over 60y or overweighted or Asians. With the increase of the elderly population, prevention of bone loss and associated fracture is becoming more important, our present data might be useful to provide dietary suggestions for people who are at higher risk of bone loss or fracture. Of course, large-scale prospective cohort studies and randomized controlled trials are need to be verified our results in further.

## MATERIALS AND METHODS

### Literature search

We searched articles aimed to evaluate the association between circulating or dietary carotenoids and fracture risk published throughout May 31th, 2016 from MEDLINE and EMBASE databases. We searched the terms of “carotenoids”, “carotene”, “lycopene”, “tomato”, “β-cryptoxanthin”, “lutein”, “zeaxanthin”, and “vegetable and fruit” on MeSH term or in the Title/Abstract of articles; and then we searched “BMD”, “osteoporotic fractures”, “osteoporosis”, and “fracture” on MeSH term or in the Title/Abstract of articles. And then we combined the 2 search results by using OR. All searches were limited to for human. Additionally, we manually searched relevant articles by screening the bibliography of selected articles, relevant reviews, and meta-analyses.

### Study selection

The inclusion criteria were listed as follows: (1) prospective, case-control, or cross-sectional study; (2) the exposure of interest was dietary or circulating carotenoids; (3) the outcome was prevalence or first incidence of fracture; (4) providing the estimation of odds ratio (OR) or relative risk (RR) and its corresponding 95% confidence interval (CI) or standard error of estimations, or reporting the data to calculate these values; (5) adult participants.

### Data extraction

The following information was extracted by two authors (Jiuhong XU and Chunli SONG) independently. Extracted study characteristics were as follows: last name of the first author, publication year, study location, number of cases/participants, age, sex, BMI, range of the carotenoids, method to determine the levels of the circulating/ diet carotenoids, OR or RR estimates and 95% CIs for each category of carotenoids, and variables adjusted for in the analysis models. If a study provided multiple risk estimates for several subtypes of carotenoids or for both sexes, the risk estimates were regarded as different reports only when they were divided into different subgroups. If the study calculated RR/OR according to the percentile categories of the carotenoids levels, the risk estimate compared highest with the lowest category was extracted. If two or more risk estimates adjusted for different confounders were reported in one study, the RRs or ORs based on the full model were selected. We also tried to contact with corresponding authors of several studies for additional data.

### Assessment of the study quality

The quality of the selected studies was assessed by using the Newcastle-Ottawa scale (NOS) [43]. The study was defined as having a high quality if the total score was no less than 6.

### Statistical methods

To assess the association between carotenoids and fracture risk, we calculated the pooled risk estimates and their 95% CIs using a random-effects model. OR was an approximate estimation for RR, because hip fracture is relatively rare. Therefore, we combined OR from case-control studies and RR from prospective studies in this meta-analysis. We performed *Q*-test and calculated I^2^ statistic to examine the between-study heterogeneity. A *P-value* for *Q*-test of < 0.10 or a I^2^ of > 50% indicated significant between-study heterogeneity [44]. Meta regression was conducted to investigate the source of heterogeneity. Subgroup analyses stratified by sex, age, BMI, different study location, and subtypes of carotenoids were conducted to investigate potential effect modifiers.

Sensitivity analyses were performed by deleting one study at each analysis to explore the source of heterogeneity and to testify the robustness of the pooled risk estimate. Potential publication bias was tested by using funnel plots, Begg's test, and Egger's test; and a *P* < 0.05 indicated a significant publication bias [45]. All analyses were conducted by using STATA 11.0 (Stata Corp). A *P* < 0.05 indicated statistical significance.
